# An efficient negative selection marker for *Mos1* -mediated single-copy integration in *Caenorhabditis elegans*

**DOI:** 10.17912/micropub.biology.000647

**Published:** 2022-10-20

**Authors:** Kandan Revathi, Kuppuswamy Subramaniam

**Affiliations:** 1 Department of Biotechnology, Indian Institute of Technology Madras, Chennai, Tamil Nadu, India

## Abstract

*Mos1* -mediated single-copy integration (MosSCI) in *C. elegans* relies on the introduction of plasmid constructs into the germ line. Such plasmids form extrachromosomal arrays containing multiple copies of the transgene. Presently, one positive-selection and four negative-selection reporters are used to identify animals that carry the integrated transgene but not the array. Even with four reporters, the negative selection is inefficient. Here, we show that the expression of the toxic protein PEEL-1 from a transgene containing the endogenous *peel-1* introns kills all array-carrying animals, which facilitates efficient selection of animals carrying the integrated transgene.

**Figure 1. Expression of peel-1 with its endogenous introns kills all array-positive animals:**
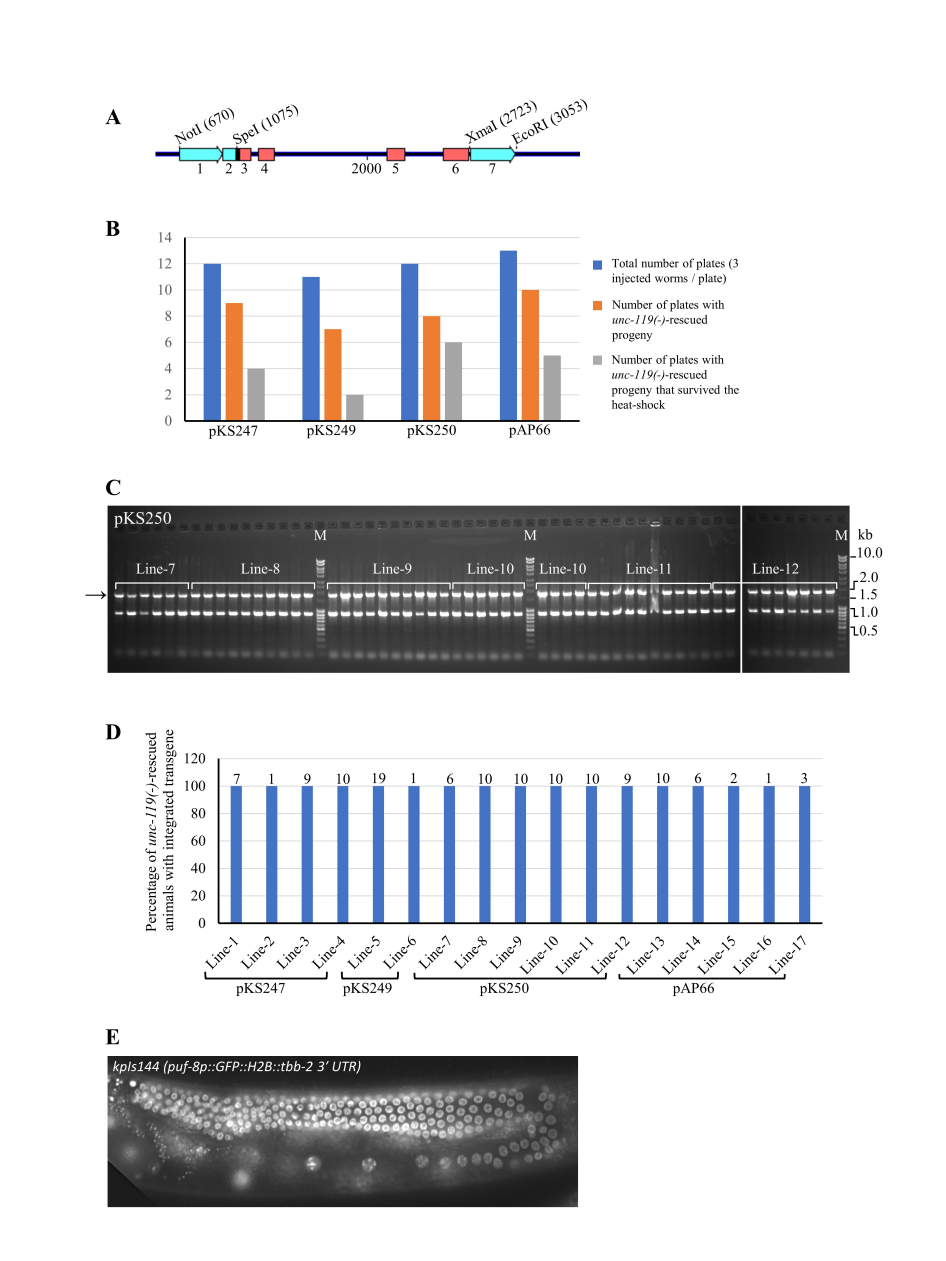
(A) Cartoon diagram of the *peel-1* expression cassette. 1. *hsp-16.41* promoter, 2. *hsp-16.41* 5′ UTR, 3‒6. *peel-1* exons, and 7. *tbb-2* 3′ UTR. The restriction enzymes used are indicated above, with their respective positions in parentheses. The blue lines between exons represent the introns. Note: Intron-1 was not included; instead, the last 16 nucleotides of exon-1, which contains the last six nucleotides of *peel-1* 5′ UTR and the first 10 nucleotides of the coding region, were included in the PCR primer used for amplifying the rest of *peel-1* coding region. (B) Metrics for the four microinjection experiments. Names of the transgenes are given below the corresponding bars. (C) Results of PCR amplification for the 55 animals tested for pKS250. In this experiment, the left integration junction was amplified using primers KS6455 and KS657. The expected 1.8-kb band is indicated by the arrow on the left. The second band about 0.9 kb seen in all lanes is due to non-specific amplification. M – DNA molecular weight markers. (D) From plates represented by the grey bars in (B), wildtype (rescued for the *unc-119* mutation) animals were cloned. All such animals in each plate were assumed to be the descendants of a single animal with successful transgene integration. Based on this, each plate was assumed to represent one independent line. The number of wildtype animals recovered and cloned from each line is mentioned above the corresponding bar. In some plates (Line-2, Line-6, and Line-16), only one wildtype animal was found to have survived the heat-shock. After collecting a few embryos, the cloned animals were subjected to PCR using integration-specific primers to determine the presence of the integrated transgene. (E) A representative image showing transgene expression in the germ line.

## Description

In *C. elegans* , *Mos1* transposition has been successfully coopted for the insertion of single copies of transgenes at predetermined chromosomal loci (Frokjaer-Jensen et al. 2008; Frokjaer-Jensen et al. 2012). This method, known as *Mos1* -mediated single-copy integration (MosSCI), employs *C. elegans* strains that contain the *Mos1* element at selected chromosomal loci (Vallin et al. 2012). The desired transgene is constructed on a plasmid vector such that it is flanked by sequences homologous (homology arms) to either side of the existing *Mos1* element on the chromosome. The transposase expressed from another plasmid that is introduced along with the transgene-carrying plasmid swaps the *Mos* 1 element on the chromosome with the transgene, which results in single-copy integration at the specific locus. The *C. elegans* strains used are homozygous for the *ed3* allele of *unc-119(ed3)* as well, which renders them locomotion-defective. The transgene plasmids contain a wildtype copy of *cbr-unc-119* which is cointegrated along with the transgene during the MosSCI event. This restores wildtype locomotion, and thus serves as a positive selection for successful insertion of the transgene. The plasmids are introduced by microinjection into the germ line. Plasmids thus introduced often join in tandem and form of extrachromosomal arrays which are transmitted in a non-Mendelian fashion (Stinchcomb et al. 1985). Due to this, in addition to the integrated copy, transgene expression can result from the extrachromosomal array as well. Consequently, the positive selection based on *unc-119(-)* rescue is insufficient to identifying animals that carry the integrated transgene but array-negative. To circumvent this, additional plasmids that carry transgene reporters without the homology arms are included for negative selection—only the array-positive animals express these reporters (Frokjaer-Jensen et al. 2008).

Currently, the negative selection comprises two stages. In stage 1, a toxic protein is expressed under the control of either a heat-shock- or a drug-dependent promoter (Frokjaer-Jensen et al. 2012; El Mouridi et al. 2021). In stage 2, animals that survive the stage 1 selection are screened for expression of the mCherry fluorescent protein that is expressed from three different plasmids (Frokjaer-Jensen et al. 2008). An animal is deemed array negative only when it survives stage 1 and lacks expression of all three fluorescent reporters. We wished to improve the efficiency of negative selection by focusing on stage 1. One of the toxic proteins used is PEEL-1, which is normally expressed in sperm, and its ectopic expression causes necrotic cell death (Seidel et al. 2011). The coding region in the currently used PEEL-1 expression vector, pMA122, lacks introns (Frokjaer-Jensen et al. 2012). In eukaryotes, introns substantially elevate gene expression by influencing the rate and/or efficiency of multiple stages of gene expression [reviewed in (Shaul 2017)]. Based on this, we reasoned that the inefficiency at stage 1 probably results from weak PEEL-1 expression that is insufficient to effectively kill the array-positive animals. To test this, we constructed a new vector, pKS235, in which the *peel-1* coding region includes all its endogenous introns except intron-1. Like pMA122, the *peel-1* coding region in pKS235 is flanked by the *hsp-16.41* promoter and the *tbb-2* 3ʹ UTR for heat-shock-inducible expression (Figure 1A).

To test if pKS235 kills array-carrying animals, we microinjected it along with pRF4, which confers dominant roller phenotype, into wildtype adult germ lines (Mello et al. 1991). Offspring of injected animals displaying roller phenotype where selected as array-carrying animals and were heat-shocked to induce PEEL-1 expression (see **Methods** ). All rollers died by about 15 hours after the heat-shock (0±0% surviving animals; n = 100 animals / replicate; total three replicates). Next, we used pKS235 as the sole negative selection marker in actual MosSCI experiments. In all, we performed four such experiments, each one to introduce a different transgene, and generated a total of 17 independent lines (Figure 1B-D). We placed three injected *unc-119(-)* animals per plate and considered all *unc-119(-)* -rescued progeny in a plate as descendants of a single integration event, although it is possible that more than one injected animal produced transgene-carrying progeny in the same plate. About 70% of plates—ranged from 64% to 77% among the four experiments—contained *unc-119(-)-* rescued animals. No *unc-119(-)* -rescued animals survived the heat-shock in 50% of these plates (range 29% to 75%). We cloned (transferred one animal / fresh plate) a maximum of 10 *unc-119(-)* -rescued animals that survived the heat-shock from each plate (except for line-5 where we cloned 19 animals). We thus cloned 124 animals from the 17 plates that had *unc-119(-)* -rescued animals.

To test if the cloned animals carried the transgene at the intended locus, we performed PCR amplification using primers that flank the left insertion junction—one primer corresponding to a chromosome position upstream of the left homology arm and another to a position within *unc-119* cassette (Figure 1C). As shown in figure 1D, the insertion efficiency was 100% for the four transgenes that we have tested (n = 124 animals from 17 lines). We confirmed the insertion of the entire transgene through PCR-amplification of the right junction as well for at least one line per transgene. Since all four transgenes were expected to express the GFP reporter (see **Reagents** ), we examined about 20 wildtype progenies of each cloned animal for GFP fluorescence. For the transgenes pKS247, pKS249 and pKS250, all progenies of all lines were positive for GFP expression. Presumably due to promoter insufficiency, no progeny of the five lines carrying the pAP66 transgene were GFP-positive, although the entire transgene was found to be correctly inserted in these lines. We checked the inheritance patterns of *kpIs143* , *kpIs144* and *kpIs145* (the transgene alleles of pKS247, pKS249, and pKS250, respectively), in one line per transgene, and found that the GFP expression followed Mendelian inheritance in each case as would be expected for an integrated single-copy transgene. Together, these observations confirm successful single-copy integration in all lines tested.

In conclusion, our results demonstrate that the intron-containing *peel-1* expression vector pKS235 allows very efficient MosSCI integration using only a single negative selection marker, obviating the need for four plasmids and a fluorescence dissection microscope for screening.

## Methods

**Strains**

The *C. elegans* strains N2, CGC43 and EG6699 used here were maintained as described (Brenner 1974). The EG6699 strain contains the *ttTi5605* Mos1 transposon insertion at 0.77 map position in chromosome II and carries a loss-of-function mutation ( *ed3* ) in *unc-119* but carries the *unc-119(-)-* rescuing *oxEx1578* extrachromosomal array (Frokjaer-Jensen et al. 2012). Animals lacking the array are homozygous for *unc-119(ed3)* and locomotion defective. Such *unc-119* -mutant animals were maintained on lawns of the *E. coli* strain HB101 grown on NGM agar plates at 20 °C and used for transgenesis.

**Plasmid constructs**

The *peel-1* expression plasmid, pKS235 was constructed as follows: 398 bp of the genomic region immediately upstream of the *hsp-16.41* start codon was PCR-amplified from the wildtype *C. elegans* genomic DNA using primers KS6484 and KS6485 and inserted between *Not* I and *Spe* I sites of Bluescript KS+. In the resulting plasmid, the genomic region corresponding to *peel-1* coding sequence, obtained by PCR-amplification using primers KS6486 and KS6487, was inserted between *Spe* I and *Xma* I sites. The *peel-1* PCR product excludes intron-1. The last 16 bp of the short *peel-1* exon-1 were included in KS6486. In the resulting plasmid, the *tbb-2* 3ʹ UTR, obtained by PCR-amplification using primers KS6488 and KS6489, was inserted between *Xma* I and *Eco* RI sites. The transposase-expressing plasmid pCFJ601 and the pCFJ350 vector for transgene insertion at the *ttTi5605* locus have been described earlier (Frokjaer-Jensen et al. 2012). The transgene-carrying plasmids, pAP66, pKS247, pKS249 and pKS250, were generated by inserting PCR fragments at the appropriate restriction sites in the pCFJ350 vector (see **Reagents** ). The promoters, 3ʹ UTRs and the H2B coding sequence were amplified from wildtype *C. elegans* genomic DNA and the GFP fragment was amplified from the *C. elegans* strain CGC43. The PCR primers used are listed below in the **Reagents** section.

**Transgenesis**

We prepared 40 μl of injection cocktail containing 50 ng/μl each of pCFJ601, pKS235 and the transgene-carrying plasmid (pAP66, pKS247, pKS249 or pKS250) in water. To remove any particulate matter that might clog the injection needle, we centrifuged the injection cocktail at 12000 g for 10 minutes and gently transferred about 30 μl to a fresh tube from the top part of the solution. To enhance the survival rate following microinjection, we injected into only one gonad. Microinjection was performed as described earlier (Mello et al. 1991). Typically, we injected about 30 to 36 young adults per transgene. Injected animals were transferred, three animals per plate, to fresh HB101 plates and incubated at 25 °C. Presence of wildtype [ *unc-119(ed3)-* rescued] progeny on the third day confirmed successful injection. Plates with wildtype animals were shifted seven days after injection to 34 °C for three hours to induce *hsp-16.41* -driven *peel-1* expression and then shifted back to 25 °C. About 15 hours (overnight) later, about 10 L1-L3 larvae displaying wildtype locomotion were picked from each plate and cloned on fresh OP50 plates and incubated at 25 °C till they reached adulthood. After they laid some embryos, each of the cloned animals were lysed in 5 μl of lysis buffer (10 mM Tris-HCl, pH 8.3 / 50 mM KCl / 1.5 mM MgCl _2_ / 0.45% Tween-20 / 0.45% Igepal CA-630) by incubation at 65 °C for 45 minutes followed by 95 °C for 15 minutes. The total lysate was used as the template for PCR amplification using primers KS6455 and KS6457, which flank the left insertion site: KS6455 corresponds to the chromosomal portion upstream of the insertion site and KS6457 to a position near the start of the *unc-119(-)* -rescuing cassette in the transgene plasmid. To confirm insertion of the entire transgene cassette, we carried out PCR using primers that flank the right insertion site as well in the lines selected based on the transgene expression.

**Fluorescence microscopy**

Live animals were anesthetized in 2 mM levamisole and observed using a Zeiss Axio Image M2 fluorescence microscope and imaged using a Zeiss Axiocam506 Mono CCD camera.

## Reagents

***C. elegans* strains**

**Table:** 

**Strain name**	**Genotype**
N2	Wildtype strain
CGC43	*unc-4(e120)/mnC1 [dpy-10(e128) unc-52(e444) umnIs32]* II
EG6699	*ttTi5605 II; unc-119(ed3) III; oxEx1578*
IT1483	*kpIs143 [mex-5p::GFP::H2B::pas-1 3ʹ UTR] II; unc-119(ed3) III*
IT1486	*kpIs144 [puf-8p::GFP::H2B::tbb-2 3ʹ UTR] II; unc-119(ed3) III*
IT1487	kpIs145 [mex-5p::GFP::H2B::pas-1 3ʹ UTR*] II; unc-119(ed3) III

**Plasmids used**

**Table:** 

**Plasmid name**	**Reference**
pCFJ601	(Frokjaer-Jensen et al. 2012)
pCFJ350	(Frokjaer-Jensen et al. 2012)

**Transgenes**

**Table:** 

**Plasmid used**	**Transgene**
pKS235	*hsp-16.41p::GFP::H2B::unc-54 3′ UTR* . Available from Addgene, deposit number 81656; Addgene ID 191855
pKS247	*kpIs143 [mex-5p::GFP::H2B::pas-1 3ʹ UTR]*
pKS249	*kpIs144 [puf-8p::GFP::H2B::tbb-2 3ʹ UTR]*
pKS250	kpIs145 [mex-5p::GFP::H2B::pas-1 3ʹ UTR*]
pAP66	*kpIs146 [W10D9.6p::W10d9.6::GFP::W10D9.6 3ʹ UTR]*

*Carries mutations in the *3ʹ UTR.* The effects of these mutations on expression will be described elsewhere.

**PCR primers**

**Table:** 

**Primer name**	**Sequence**	**Description**
KS6484	TCTGCAGCGGCCGCGATCACCAAAAACGGAACGT	Forward, for *hsp-16.41* promoter + 5ʹ UTR
KS6485	TCTGCAACTAGTTTTCGAAGTTTTTTAGATGCACTAGAACAAAGCG	Reverse, for *hsp-16.41* promoter + 5ʹ UTR
KS6486	TCTGCAACTAGTACAAGGATGCGCTTTGATTTCCAAAACTTAAAATTTTCGATGATTTTCATATTTTTGTGGAACATCATAGTCGG	Forward, for *peel-1* coding sequence (CDS)
KS6487	TCTGCACCCGGGTCATGGATTTTCAACACTTGGATC	Reverse, for *peel-1* CDS
KS6488	TCTGCACCCGGGATGCAAAATCCTTTCAAGCA	Forward, for *tbb-2* 3' UTR
KS6489	TCTGCAGAATTCGACTTTTTTCTTGGCGGCAC	Reverse, for *tbb-2* 3' UTR
KS4921	TCTGCACTTAAGTCCCATGCCCACTGGCTCAGTCACATGAG	Forward, *W10D9.6* promoter + CDS with *Afl* II site
KS4781	TCTGCAAGATCTAATTTTCAGTCCATTTTTCATGTGATCG	Reverse, *W10D9.6* promoter + CDS with *Bgl* II site
KS5419	TCTGCAGCGGCCGCTGAATAAATTCCTTTTTTTTTTGTTGATCTCTGAGTATTTAGAAAACTGC	Forward, *W10D9.6* 3' UTR with *Not* I site
KS5420	TCTGCAGCGGCCGCAGGGTACTAGTCCTAAATC	Reverse, *W10D9.6* 3' UTR with *Not* I site
KS6643	TCTGCACTTAAGCAGAGACAACCATCTGCAAG	Forward, *mex-5* promoter with *Afl* II site
KS6644	TCTGCAAGATCTATTTGATGCCGCTTTCATTCTCTGTCTG	Reverse, *mex-5* promoter with *Bgl* II site
KS6645	TCTGCAAGATCTATGAGTAAAGGAGAAGAACTTTTCACTGGA	Forward, GFP CDS with *Bgl* II site
KS6646	TCTGCAAGATCTCCTTTGTATAGTTCATCCATGCCATGTG	Reverse, GFP CDS with *Bgl* II site
KS6647	TCTGCAACTAGTCCACCAAAGCCATCTGCCAA	Forward, H2B CDS with *Spe* I site
KS6648	TCTGCAACTAGTGCGGCCGCTTACTTGCTGGAAGTGTACTTGG	Reverse, H2B CDS with *Not* I and *Spe* I sites
KS6649	TCTGCAGCGGCCGCAATCAAATCGCCAACCGAG	Forward, *pas-1* 3'UTR with *Not* I site
KS6650	TCTGCAGCGGCCGCCCTTTGCAGAGACTCTTTC	Reverse, *pas-1* 3'UTR with *Not* I site
KS6686	TCTGCACTTAAGGGAAATCACCTTTCCCGAGT	Forward, *puf-8* promoter with *Afl* II site
KS6687	TCTGCAAGATCTCGGACGACTCATCAGGAACG	Reverse, *puf-8* promoter with *Bgl* II site
KS6688	TCTGCAGCGGCCGCATGCAAGATCCTTTCAAGCATTC	Forward, for *tbb-2* 3' UTR with *Not* I site
KS6689	TCTGCAGCGGCCGCTGAGACTTTTTTCTTGGCGGCAC	Reverse, for *tbb-2* 3' UTR with *Not* I site
KS6455	TCTGGCTCTGCTTCTTCGTT	Forward, in chromosome II upstream of the left homology arm of pCFJ350
KS6457	CAATTCATCCCGGTTTCTGT	Reverse, within *unc-119* cassette
